# Improving protein function prediction methods with integrated literature data

**DOI:** 10.1186/1471-2105-9-198

**Published:** 2008-04-15

**Authors:** Aaron P Gabow, Sonia M Leach, William A Baumgartner, Lawrence E Hunter, Debra S Goldberg

**Affiliations:** 1 Department of Pharmacology, University of Colorado at Denver and Health Sciences Center, MS 8303, RC-1 South, 12801 East 17th Avenue, L18-6101, PO Box 6511, Aurora, CO 80045, USA; 2 Department of Computer Science, University of Colorado at Boulder, Boulder, CO 80309, USA; 3 Department of Electrical Engineering (ESAT), Research Division SCD, Katholieke Universiteit Leuven, B-3001 Leuven, Belgium

## Abstract

**Background:**

Determining the function of uncharacterized proteins is a major challenge in the post-genomic era due to the problem's complexity and scale. Identifying a protein's function contributes to an understanding of its role in the involved pathways, its suitability as a drug target, and its potential for protein modifications. Several graph-theoretic approaches predict unidentified functions of proteins by using the functional annotations of better-characterized proteins in protein-protein interaction networks. We systematically consider the use of literature co-occurrence data, introduce a new method for quantifying the reliability of co-occurrence and test how performance differs across species. We also quantify changes in performance as the prediction algorithms annotate with increased specificity.

**Results:**

We find that including information on the co-occurrence of proteins within an abstract greatly boosts performance in the Functional Flow graph-theoretic function prediction algorithm in yeast, fly and worm. This increase in performance is not simply due to the presence of additional edges since supplementing protein-protein interactions with co-occurrence data outperforms supplementing with a comparably-sized genetic interaction dataset. Through the combination of protein-protein interactions and co-occurrence data, the neighborhood around unknown proteins is quickly connected to well-characterized nodes which global prediction algorithms can exploit. Our method for quantifying co-occurrence reliability shows superior performance to the other methods, particularly at threshold values around 10% which yield the best trade off between coverage and accuracy. In contrast, the traditional way of asserting co-occurrence when at least one abstract mentions both proteins proves to be the worst method for generating co-occurrence data, introducing too many false positives. Annotating the functions with greater specificity is harder, but co-occurrence data still proves beneficial.

**Conclusion:**

Co-occurrence data is a valuable supplemental source for graph-theoretic function prediction algorithms. A rapidly growing literature corpus ensures that co-occurrence data is a readily-available resource for nearly every studied organism, particularly those with small protein interaction databases. Though arguably biased toward known genes, co-occurrence data provides critical additional links to well-studied regions in the interaction network that graph-theoretic function prediction algorithms can exploit.

## Background

The putative characterization for unknown proteins has traditionally relied on sequence homology, for example as assessed by BLAST score. This approach is inadequate for proteomic-wide function identification as it has a failure rate of 20–40% in newly sequenced genomes [1]. Sources of error stem from difficulties in determining the correct homologue, evolutionary divergence of function and even the lack of annotated homologues [2].

New methods for proteomic-scale function prediction which do not rely on sequence homology draw from high-throughput data to make inferences, including several techniques that use protein-protein interaction graphs [1,3-8]. Protein function is predicted based on the functions assigned to a protein's neighbors in the interaction graph, using either a simple majority vote of the functions assigned to the immediate neighbors [3] or propagating functional assignments through a more global neighborhood [5-7].

Previous research has significantly improved algorithmic performance by integrating more diverse interaction types, such as gene expression data, genetic interactions, or phylogenetic based features [6,9,10]. Including additional sources affects the graph topology and its correctness, whether by joining previously disconnected areas of the graph, reinforcing support for existing edges or potentially adding false positives. Thus, one obvious question becomes how useful is each of these sources to a graph-theoretic function prediction algorithm.

This paper examines closely the use of Medline literature abstracts as a potential high-throughput annotation source. Literature offers a valuable resource as the original and historical source of information contributed by biologists; however, much of that information has yet to be human-curated in a computable form. Moreover, the current pace of paper submissions outstrips curators' ability to extract information. Thus, a popular trend is to develop sophisticated natural language processing methods to mine free-text data for protein relationships. Previously, various researchers have tried directly mining text for functional information [11-17], yet the accurate automatic characterization of protein interaction remains a challenge for current systems [18,19].

In this paper, we consider the straight-forward approach of mining for potential interactions by identifying co-occurrence of protein names in Medline abstracts, similar to the use of co-occurrence data by Schlitt [20] as a way of validating possible protein-protein interactions. The caveats of such derived relationships are a strong bias towards previously studied proteins and a potentially high false positive rate owing to the difficulty of identifying protein names and mapping them to the appropriate identifiers [18]. Despite these issues, the readily available co-occurrence data can be a valuable supplement to protein-protein interaction data for graph-theoretic function prediction algorithms. Medline abstracts describe many different relationships apart from protein-protein interactions, such as shared pathway membership, co-regulation, genetic interactions and structural similarity, that can inform function prediction. Observed co-occurrence may also highlight previously uncharacterized relationships. In organisms where there are very few protein-protein interactions reported in the public databases, co-occurrence data can provide critical linkages among otherwise sparsely-connected annotated regions of the protein interaction graph.

While we could further combine co-occurrence data with other interaction information, here we purposefully isolate the literature data source and quantify its individual contribution as a supplement to protein interaction data. The originality and benefit of our study is many-fold. Firstly, to our knowledge, there has been no systematic study of the use of literature-based information in graph-theoretic function prediction algorithms. While systems such as STRING [10], PubGene [15], CoPub [17], Prolinks [21] and iHoP [22] use co-occurrence information to infer pairwise functional linkages between proteins, those efforts do not report function prediction results using graph-theoretic algorithms applied to networks constructed from those linkages. Since graph-theoretic approaches can consider the global network structure, these approaches are especially powerful given the tendency for literature to describe relationships over disjoint subsets of high-studied proteins.

Secondly, nearly all previous demonstrations of function prediction have been benchmarked on a single organism, typically yeast. Even when algorithms use multi-organism data to make predictions, particularly for prokaryotes (e.g. [23,24]), the majority still only report evaluations on a single organism. Exceptions include, for example [25,26]. Focusing on eukaryotes, we provide results using identical experimental settings for not only yeast but worm and fly, both of which have sparser coverage than yeast in functional annotation and interaction databases. Thirdly, the simplest use of co-occurrence data asserts an interaction between proteins mentioned together at least once (or twice) in literature abstracts. We investigate the performance of prediction algorithms when that requirement becomes more stringent, moving beyond a simple binary indicator to requiring an interaction surpass a confidence threshold before asserting it as true. In addition to comparing two quantitative methods from the literature based on the hypergeometric distribution [21,27] and mutual information [17] which are symmetric in bias with respect to a given protein pair, we introduce an asymmetric confidence score which encodes a bias for the number of co-mentions relative to mentions for an individual protein and argue its preferred relevance to this application.

Finally, we explore several parameters that affect graph-theoretic function prediction algorithms. Nabieva *et al*. [7] consider yeast annotation at Level 2 of the MIPS Functional Classification hierarchy [28]. We supplement their analysis by considering algorithm performance as the granularity for a given assignment increases, comparing to using Level 3 in MIPS. We also consider the effect of using different gold-standard annotation sources by comparing the yeast results using MIPS to those using Biological Process and Molecular Function SLIM terms from the Gene Ontology (GO) [29]. Nabieva *et al*. also experimented with GO and report that using GO annotations does not affect their overall conclusions; we provide a complete side by side comparison in order to highlight differences in coverage and informativeness when using these annotation sources.

## Methods

### Extracting protein names from Medline abstracts

For each species, all Medline [30] abstracts that contained keywords for the common and Latin species names in the abstract, title or MeSH header were collected. Text strings referring to a gene (or gene family) were tagged using LingPipe [31] which takes as input individual blocks of text, called tokens, representing a word, number or symbol. The tagger extracts gene mentions from text using a hidden Markov model (HMM) trained on a corpus of sentences manually annotated for gene mentions [32]. There are eight states in the HMM representing the beginning, middle and end tokens of a gene mention as well as the surrounding tokens and those that are not part of gene mentions. The instances of gene mentions are then mapped to Entrez Gene identifiers after canonicalization by lowercasing, removing punctuation and whitespace and replacing Roman numerals with Arabic numerals [33]. If a single text string mapped to multiple Entrez identifiers, such as glycosyltransferase or C Type lectin, a separate instance of co-occurrence was asserted from each of those Entrez identifiers. To alleviate false positives resulting from abstracts that mention a very large number of proteins or contain text which maps to non-specific gene names such as pseudogene, any data from Medline abstracts that resolve to more than 100 Entrez identifiers was removed.

### Determining co-occurrence

Typical applications of literature co-occurrence assert an interaction if the pair of proteins is mentioned at least once (or twice) in a Medline abstract. However, this method is prone to false positives and a more rigorous method should be used that quantifies the strength in belief of the literature association. Jenssen *et al*. [15] examined the accuracy and type of interactions found among genes mentioned more than once or more than five times together and found a decrease in the number of false positives as the number of co-occurrences increased. Extensions have been used to assess confidence of co-occurrence using mutual information [17] and the hypergeometric distribution [21,27] measures. Other alternatives might be to use the Normalized Google Distance [34], term frequency inverse document frequency (TF-IDF) [35] or a z-score like method [36]. We evaluate the two methods based on mutual information and the hypergeometric distribution in our comparisons. Let *n*_*x *_be the number of documents retrieved from Medline citing protein *x*, *n*_*xy *_be the number of documents that cite both proteins *x *and *y *and *N *be the total number of Medline abstracts. Then the relative mutual information score [17] between proteins *x *and *y *(normalized to the range 0–1, with 1 meaning high confidence) is

$$MUT(x,y)=0.01+0.99\frac{R-R_{\min}}{R_{\max}-R_{\min}}$$

where *R *= log_10_([*n*_*xy*_*N*]/[*n*_*x*_*n*_*y*_]) and *R*_min _and *R*_max _are the lowest and highest *R *values over all pairs *x *and *y*. The hypergeometric score [27] calculates the probability that the number of co-citing abstracts is greater than or equal to a value *l *as

$$p(n_{xy}>=l|n_{x},n_{y},N)=1-\sum_{k=0}^{l-1}p(k|n_{x},n_{y},N)$$

where *p*(*k *| *n*_*x*_, *n*_*y*_, *N*) is the hypergeometric distribution:

$$p(k|n_{x},n_{y},N)=\frac{\left(\begin{array}{c} n_{x}\\ k \end{array}\right)\left(\begin{array}{c} N-n_{x}\\ n_{y}-k \end{array}\right)}{\left(\begin{array}{c} N\\ n_{y} \end{array}\right)}.$$

This value computes the significance of co-occurrence, such that smaller numbers mean the observed co-occurrence is less likely by chance, i.e. low numbers mean high confidence. To scale the values to be analogous to the other measures, where high values mean high confidence edges, we define the hypergeometric-based co-occurrence measure as

*HY G*(*x*, *y*) = 1 - *p*(*n*_*xy *_>= *l *| *n*_*x*_, *n*_*y*_, *N*).

One potential disadvantage of these measures for our application is the fact that each is symmetric with respect to the contribution of the protein pair and does not emphasize the frequency of co-occurrence relative to the individual occurrence frequencies. For example, a low co-occurrence frequency of two proteins *x *and *y *becomes significant when the individual protein occurrence frequency of *x *is also low since it implies that *x*, though hardly mentioned, is always mentioned in conjunction with *y*. This is true regardless of the occurrence frequency of a protein *y*.

An asymmetric measure is important for biology applications since some genes are more well studied than others, and there is particular interest in genes with little prior information. For this reason, we define the *Asymmetric Co-occurrence Fraction (ACF) *which includes a bias towards genes mentioned infrequently:

$$ACF(x,y)=\frac{n_{xy}}{\min[n_{x},n_{y}]}.$$

Mathematically speaking, the measure itself is symmetric, since *ACF*(*x*, *y*) = *ACF*(*y*, *x*) yet we label the measure as asymmetric to emphasize the bias towards less characterized proteins. We compare our new measure to the two alternatives, *MUT *and *HY G*, to investigate whether important relationships are overlooked under symmetry. To reduce false positives for any of the three co-occurrence based measures, we add the additional requirement that *n*_*xy *_> 1, otherwise the co-occurrence value is set to zero.

Regardless of the co-occurrence measure used, the method for handling ambiguity in normalizing gene names artifactually inflates the values within gene families. For example, the text string clec resolved to 83 distinct Entrez identifiers of C Type lectin genes in worm, resulting in all pairwise interactions among those genes, all with weights of 1.0 regardless of the co-occurrence measure. The consequence is that at high co-occurrence thresholds, the graphs contain many clusters representing gene families [see Additional files 1, 2 and 3].

### Interaction dataset creation

Protein-protein interactions were extracted from the Database of Interacting Proteins (DIP) [37] for yeast, worm and fly according to the publication or experiment type as given in Table 1. Genetic interactions for yeast were also taken from DIP as referenced by the publications in Table 1, while the genetic interactions for worm and fly were taken from WormBase and FlyBase (see Availability and requirements section for URL). For each of the three co-occurrence measures, eleven datasets of edges were extracted by fixing a threshold in increments of 0.1 and including all edges where the confidence value assigned by the measure meets or exceeds the threshold. These datasets are referred to by the threshold fraction, e.g. *ACF *≥ 0.3.

**Table 1:** Physical and Genetic Correspondence to Annotation

**PHYSICAL**									
**Yeast**	**MIPS**	**MF**	**BP**	**Worm**	**MF**	**BP**	**Fly**	**MF**	**BP**
Uetz 1498	37	15	32	Y2H 2619	10	6	Y2H 20045	10	14
Ito 4469	19	8	17	Aff Chr 26	19	0	Immunoblotting 2	100	100
Fromont 175	26	15	37				Immuno Prec 5	60	100
Gavin 3139	67	41	70				Gel Retardation 2	100	100
Ho 3464	38	15	36				Experimental 1	100	100
							Biophysical 3	100	100
							Alanine Scanning 2	100	100

**GENETIC**									
**Yeast**	**MIPS**	**MF**	**BP**	**Worm**	**MF**	**BP**	**Fly**	**MF**	**BP**

Bellaoui 34	79	0	82	All 20543	42	50	All 6523	32	69
Davierwala 564	40	13	39						
Huang 58	55	5	55						
Goehring 63	65	5	61						
Kozminski 30	70	7	53						
Krogan 36	50	11	78						
Parsons 86	55	2	43						
Tong 5907	49	12	39						

Percentage of edges in the full graph which connect proteins sharing the same annotation according to the gold standard. These values are the *r*_*i *_used in the calculation of edge weights by the noisy-or function. The number of edges scored is shown following the experimental group name. Listed by first author, PubMed identifiers for the groups are: Uetz PMID:10688190, Ito PMID:10655498, PMID:11283351, Fromont PMID:9207794, Gavin PMID:11805826, Ho PMID:11805837, Bellaoui PMID:12912927, Davierwala PMID:16155567, Huang PMID:12077337, Goehring PMID:12686605, Kozminski PMID:12960420, Krogan PMID:12773564, Parsons PMID:14661025, and Tong PMID:14764870. Abbreviations: GO SLIM Molecular Function (MF), GO SLIM Biological Process (BP), yeast two-hybrid (Y2H), affinity chromatography (Aff Chr), immunoprecipitation (Immuno Prec).

### Functional annotation and evidence type scoring

Yeast proteins were annotated using the MIPS [28] functional catalog at Levels 2 and 3 in the hierarchy. Yeast, fly and worm were annotated to their Molecular Function and Biological Process SLIM terms in the Gene Ontology(GO) [29] using the generic SLIM ontology for fly and worm and the yeast-specific SLIM ontology for yeast; all are available from the GO website [38]. The genes were mapped from the full-ontology gene association file to the GO SLIM terms using the program map2slim.perl script also available from the GO website. According to Ofran *et al*. [2], predicting GO classification is now becoming the standard in the field of automated function prediction. Though the Biological Process ontology of GO more closely matched the MIPS categories in semantics, Molecular Function was also included as it offered comparable coverage to Biological Process in yeast and fly while offering much greater coverage in worm. Under either MIPS or GO SLIM, a protein can be assigned to more than one category.

Following the approach of Nabieva *et al*. [7] and the STRING database [10], the functional annotation source was used to evaluate the quality of each interaction data set. The quality score of each interaction source, separated by publication or experiment type as given in Table 1 and 2, was based on the percentage of all interactions from the group that link two proteins assigned to the same category by the annotation gold standard. For example, approximately 37% of interactions from the Uetz dataset connect two proteins with shared MIPS functions, giving each interaction from the Uetz dataset a reliability score of *r*_*Uetz *_= 0.37. Interactions supported by more than one source received a combined weight based on the scores of the individual contributors, using the noisy-or function [7,10]. The noisy-or edge weight was computed as 1 - ∏_*i*_(1 - *r*_*i*_) where the product was taken over all experiment groups *i *where the interaction was found and *r*_*i *_was the corresponding reliability of group *i*. Thus, if the Gavin experimental group had a protein-protein interaction that was also in Uetz, the MIPS Gavin evidence score of *r*_*Gavin *_= 0.67 was combined with the *r*_*Uetz *_= 0.37 for Uetz using noisy-or to increase the final score for the edge strength. When using MIPS, an annotation was shared if for the depth of annotation examined, both proteins had identical annotation, so YOR396W, in functional category 10.01.03.01, and YPL001W in functional category 10.01.09.05, would share an annotation at depth 2 (both are 10.01 or "DNA Processing") but would not share an annotation at depth 3. For the co-occurrence datasets created from a particular threshold, all edges which meet or exceed the threshold were considered as a single group when calculating the reliability, ignoring the actual value assigned to the edge by the co-occurrence measure.

**Table 2:** Co-occurrence Correspondence to Annotation

**Mutual Information Measure (MUT)**										
**Fraction**	**Yeast**	**MIPS**	**MF**	**BP**	**Worm**	**MF**	**BP**	**Fly**	**MF**	**BP**
> 0.0	8621	80	41	71	21847	76	57	17508	47	70
≥0.1	8615	80	41	71	21711	77	57	17422	47	70
≥0.2	8554	80	41	71	21177	78	58	16753	47	71
≥0.3	8210	80	41	71	20209	80	60	14494	49	72
≥0.4	7216	80	43	72	18811	83	63	10625	53	76
≥0.5	5592	82	46	73	17813	85	64	7021	56	76
≥0.6	3605	82	51	74	15857	91	67	4112	63	74
≥0.7	1856	82	56	74	12770	91	61	1965	59	68
≥0.8	700	77	54	72	10924	94	61	1002	56	63
≥0.9	159	65	45	75	6360	94	91	308	38	40

**Hypergeometric Measure (HYG)**										
**Fraction**	**Yeast**	**MIPS**	**MF**	**BP**	**Worm**	**MF**	**BP**	**Fly**	**MF**	**BP**

>0.0	8621	80	43	73	21847	76	57	17508	47	70
≥0.1	8614	80	43	73	21739	77	57	17125	47	71
≥0.2	8607	80	43	73	21680	77	57	17044	47	71
≥0.3	8600	80	43	73	21671	77	57	16907	47	71
≥0.4	8591	80	43	73	21397	78	58	16719	47	71
≥0.5	8572	80	43	73	21202	78	58	16575	48	71
≥0.6	8557	80	43	73	21183	78	58	16360	48	71
≥0.7	8532	80	44	73	21159	78	58	16060	48	71
≥0.8	8466	80	44	73	20650	79	59	15665	48	71
≥0.9	8368	80	44	73	20386	80	60	14764	49	72

**Asymmetric Co-occurrence Fraction Measure (ACF)**										
**Fraction**	**Yeast**	**MIPS**	**MF**	**BP**	**Worm**	**MF**	**BP**	**Fly**	**MF**	**BP**

>0.0	8621	80	41	71	21847	76	57	17508	47	70
≥0.1	6220	82	45	73	20063	80	60	9610	56	75
≥0.2	4241	82	49	74	17836	84	63	6786	58	76
≥0.3	2947	82	54	76	17353	86	63	5078	61	76
≥0.4	2283	82	56	76	17023	87	64	4178	64	77
≥0.5	1745	80	55	74	16875	87	63	3589	66	77
≥0.6	1195	78	55	73	16574	88	64	2922	68	76
≥0.7	713	78	56	72	16082	88	64	2494	70	76
≥0.8	536	74	52	69	15938	89	63	2277	71	77
≥0.9	390	68	47	65	15821	89	64	2031	72	75

Percentage of edges in the full graph which connect proteins sharing the same annotation according to the gold standard. These values are the *r*_*i *_used in the calculation of edge weights by the noisy-or function. The number of edges scored is shown in the columns labeled by organism name. Abbreviations: GO SLIM Molecular Function (MF), GO SLIM Biological Process (BP).

### Prediction algorithms

To test the performance and stability of local and global graph-theoretic approaches, the highest performing algorithms as characterized by Nabieva *et al*. (2005), Majority and Functional Flow, were examined. The Majority algorithm (as proposed by [3] and extended by [7]) predicts annotations as the weighted majority vote of annotations of adjacent nodes, using the noisy-or edge strengths as weights. Functional Flow treats each annotated protein as a source of functional flow and propagates support for each annotation of that protein across its outgoing edges, subject to the capacity of the edges as defined by noisy-or edge strengths. The algorithm iterates for a fixed number of time steps (the previously used value of six was used), using local rules to combine and propagate incoming functional flow with outgoing functional flow at each protein at each time step. The final prediction score for each annotation term at an unannotated protein node represents how much support for the function flowed through that node during execution.

### Evaluation

Performance of prediction algorithms using a given graph was measured using a modified receiver operator characteristic (ROC) curve that measures the number of true positive predictions against the number of false positive predictions as the prediction strength threshold changed. As in the previous work ([7]), when an algorithm scores more than one function annotation above the prediction strength threshold for a protein, the prediction counts as a true positive when the (strict) majority of predicted annotations are contained in the set of functions assigned by the annotation gold standard. Otherwise, the prediction counts as a false positive. Treating the annotations in MIPS or GO SLIM as a gold standard, all results use two-fold cross-validation, hiding the functional annotations for half of the known proteins and predicting the other half. Results are reported as the sum of the true and false positives from both crosses. While leaving out half of the annotations rather than using a smaller percentage or performing leave-one-out cross validation may underestimate the capability of a given prediction algorithm, the aim was to evaluate performance in the type of extreme setting that exists for less well-studied organisms or for proteins with few well-studied neighbors. This severe sparsity gives results that are more relevant to biologically interesting conditions than a more conservative setting where the prediction task is easier.

## Results

### Complementing the PPI data with co-occurrence data

Using the most general definition of co-occurrence, whereby an interaction exists between two proteins mentioned at least twice together in the literature (denoted COLIT), co-occurrence data was a significant source of interactions for all organisms (Table 3). The contribution was particularly significant for worm for which little PPI data exists relative to the number of genes (Table 3, panel 1 and 2). The COLIT graphs were denser for worm and fly (Table 3, panel 3), due in part to the presence of larger gene families in these organisms relative to yeast [see Additional files 1, 2 and 3].

**Table 3:** Characterization of Graphs

	**Source**	**Yeast**	**Worm**	**Fly**
**Number of Edges (Nodes) in Full Graph**	PPI	12177 (4581)	2619 (1955)	20056 (6689)
	GENETIC	4429 (1304)	20543 (2934)	6523 (2734)
	COLIT	8621 (2605)	21847 (1665)	17508 (2228)
**Number of Edges (Nodes) in Largest Connected Component**	PPI	12001 (4463)	2451 (1685)	19992 (6573)
	GENETIC	4427 (1301)	20359 (2736)	6418 (2551)
	COLIT	8390 (2291)	12018 (921)	17324 (2022)
**Median (Maximum) Number of Neighbors**	PPI	3 (288)	1 (145)	3 (173)
	GENETIC	2 (153)	6 (150)	2 (191)
	COLIT	4 (88)	16 (243)	8 (278)
**% Edges Intersect GENETIC**	PPI	0.09	0.9	0.2
	COLIT	1	21	6
**% Edges Intersect PPI**	GENETIC	0.2	0.1	0.6
	COLIT	7	0.07	0.7
**% Edges Only From SOURCE in PPI+SOURCE**	GENETIC	27	89	24
	COLIT	40	90	46

Various measures to characterize the density and overlap of graphs. All values are given for the largest connected component of the graph except in the first panel as indicated. Abbreviations: PPI – *only *edges from experiments measuring protein-protein interactions; GENETIC – *only *edges from genetic assays; COLIT – *only *edges between proteins mentioned at least twice together in literature abstracts.

The GENETIC and COLIT graphs had a comparable number of edges in yeast and worm, with a particularly strong overlap in worm (Table 3, panel 4). The overlap may reflect that worm gene name conventions often define genes by phenotype, *e.g*., mechanosensory abnormality (mec genes) or synthetic lethal with mec-8 (sym genes), causing co-occurrence to implicitly discover genetic relationships. The interactions offered by COLIT or GENETIC data were a significant addition to the PPI data (Table 3, panel 5 and 6), with up to 90% of the total number of edges in the combined networks contributed solely by the non-PPI source. The amount of overlap between COLIT and PPI data (7.2%) was similar to previous findings for the human protein-protein interaction map with curated interactions from the literature and yeast two-hybrid data [39].

### Characterization of annotation source

The coverage of functional annotation used in the prediction task varied depending on the type of data and the organism (Table 4). Yeast annotation sources generally had better coverage than the worm and fly sources, with around 30% uncharacterized proteins versus 40–50% in the other organisms. High throughput PPI data was a valuable source for all organisms, involving the highest percentage of uncharacterized genes (with the exception of fly), relative to the COLIT and GENETIC sources. The PPI network also showed a larger percentage of proteins completely surrounded by uncharacterized proteins. In contrast, COLIT offered the lowest percentage of interactions among uncharacterized genes, demonstrating the bias toward well-known proteins. The PPI interactions generally also had the poorest correspondence to shared function, while the COLIT interactions had strong correspondence.

**Table 4:** Characterization of Annotations

	**Source**	**Yeast MIPS**	**Yeast GO**		**Worm GO**		**Fly GO**	
			**MF**	**BP**	**MF**	**BP**	**MF**	**BP**
**Annotation Terms**		85	37	32	37	48	39	49
**Percentage Unknown Nodes**	PPI	23	38	28	41	51	39	42
	GENETIC	14	32	16	24	26	53	41
	COLIT	2	15	7	17	24	9	7
**Percentage Connected to ≥ 1 Unknown**	PPI	31	53	36	46	61	69	70
	GENETIC	14	40	24	53	50	32	17
	COLIT	4	34	18	53	59	33	28
**Percentage Only Surrounded by Unknowns**	PPI	4	9	5	17	29	15	16
	GENETIC	0.9	7	4	4	4	7	1
	COLIT	0.08	2	1	4	2	1	1
**Percent Edges Connecting Nodes Sharing Function**	PPI	37	18	36	10	6	10	14
	GENETIC	48	12	40	42	50	32	70
	COLIT	80	40	71	59	53	47	70

Various measures to characterize the completeness and connections among gold-standard annotations in the graphs. All values are given for all nodes in the Largest Connected Component of the graph. The number of nodes and edges from which these percentages are calculated are shown in panel 2 of Table 3. Unknown refers to proteins uncharacterized by the annotation source. Other abbreviations are as given in Table 3.

For the PPI and GENETIC interactions, the low-throughput assays were generally more reliable in terms of capturing functional relationships than the high throughput sources (Table 1). Also, the correspondence of these interaction sources with annotation in yeast was comparable for GO BP SLIM terms and the MIPS categories. All sources generally showed less correspondence to Molecular Function than Biological Process, with the exception of worm PPI sources.

### Comparison of co-occurrence measures

**Figure 1:**
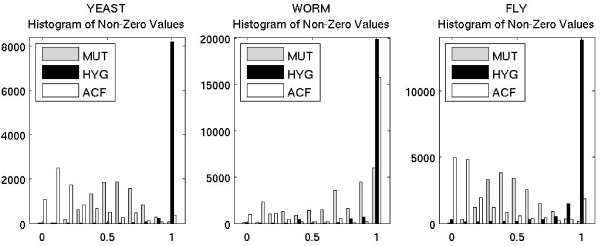
**Histogram Comparison of Co-Occurrence Measures**. Histogram of the number of proteins assigned a given confidence value by the co-occurrence measures. Abbreviations: MUT – Mutual Information Measure; HYG – Hypergeometric Measure; ACF – Asymmetric Co-occurrence Fraction.

Three measures were used to modify the definition of co-occurrence from a simple binary indicator to a qualitative assessment of interaction confidence. The Mutual Information co-occurrence measure (*MUT*) typically assigned a mid-range confidence level to the majority of edges (Figure 1) which had the consequence that there was a sharp decrease in the number of edges in the graph for thresholds greater than 0.5 (see Table 2 under organism name for graph sizes). In contrast, the Hypergeometric co-occurrence measure (*HY G*) tended to assign a high value to nearly all edges (Figure 1) which meant that the graph was nearly the same at all thresholds (Table 2). In fact, the *HY G *graphs at a threshold of 0.9 contained 97%, 93% and 84% of the number of edges in the 0.0 threshold graph for yeast, worm and fly, respectively. For the *MUT *graphs, the corresponding values were 2%, 29% and 2%, a remarkable difference. The Asymmetric Co-occurrence Fraction typically assigned a majority of the edges a lower confidence (Figure 1).

With respect to the functional annotation sources, the range of reliabilities was comparable across all three measures, regardless of organism or annotation source (Table 2). The same trends observed for PPI and GENETIC sources was observed for the co-occurrence sources. In yeast, correspondence to MIPS was again comparable to GO SLIM Biological Process. Biological Process had higher correspondence than Molecular Function in yeast and fly, but surprisingly the opposite was true in worm.

### Co-occurrence differences by asymmetry

To examine whether the asymmetry was a real factor in our application, the high scoring edges by *ACF *were compared to their *MUT *and *HY G *values to identify examples where the *ACF *assigned an edge a high score yet the other two did not. Examining particular examples offers an intuition for the differences among the measures; a more quantitative assessment appears further below.

Since *HYG *essentially assigned a high weight to all edges, high valued *ACF *edges always corresponded to high valued *HYG *edges. The major difference between *ACF *and *HYG *was seen in cases where the individual mentions were relatively high and the overlap was low. For example, in yeast where the total number of abstracts indexed was *N *= 30471 and fewer than 2% of the genes were mentioned in more than 100 abstracts, *ACF *= 0.03 while *HYG *= 1.0 when *n*_*x *_= 100, *n*_*y *_= 101 and *n*_*xy *_= 3. In this case, it is arguable whether the small overlap should indeed be seen as significant and *HYG *preferred. The difference between a high *MUT *and low *ACF *was seen in the opposite case where the number of individual mentions was small. For example, *ACF *= 0.16 and *MUT *= 0.69 for an edge in yeast between two proteins, each mentioned 12 times, with an overlap of 2. It is unclear whether *MUT *should be preferred in this case since infrequently studied proteins should be emphasized so perhaps for these cases, *ACF *does not show an advantage.

Conversely, large differences between high *ACF *and low *MUT *occur when one protein has many mentions, the second protein has only few, yet each of the mentions includes the first protein. Generally for the top differences between *ACF *and *MUT *in yeast, the *MUT *score was approximately 0.5 while the *ACF *score was 1.0. In the top three examples, one protein was mentioned more than 300 times, the other was mentioned only 2 or 5 times, and always together with the first protein. These examples included a link between GCN4 and PCL5, where the Entrez Gene description and GeneRif of PCL5 mentioned that it is specifically required for GCN4 degradation and stabilization, a link between RAD51 and MEI5, where the description and GeneRif again contain text connecting the two, and a link between HSC82 and HCH1, where interestingly Entrez Gene listed two physical binding assays which support this interaction, though neither publication was in the PPI network used here. All three examples captured real relationships between the proteins, suggesting that there is a true benefit in accounting for the asymmetry of protein mentions and that interactions assigned high *ACF *values are likely biologically correct.

### Use of Functional Flow to evaluate benefits of co-occurrence data

**Figure 2:**
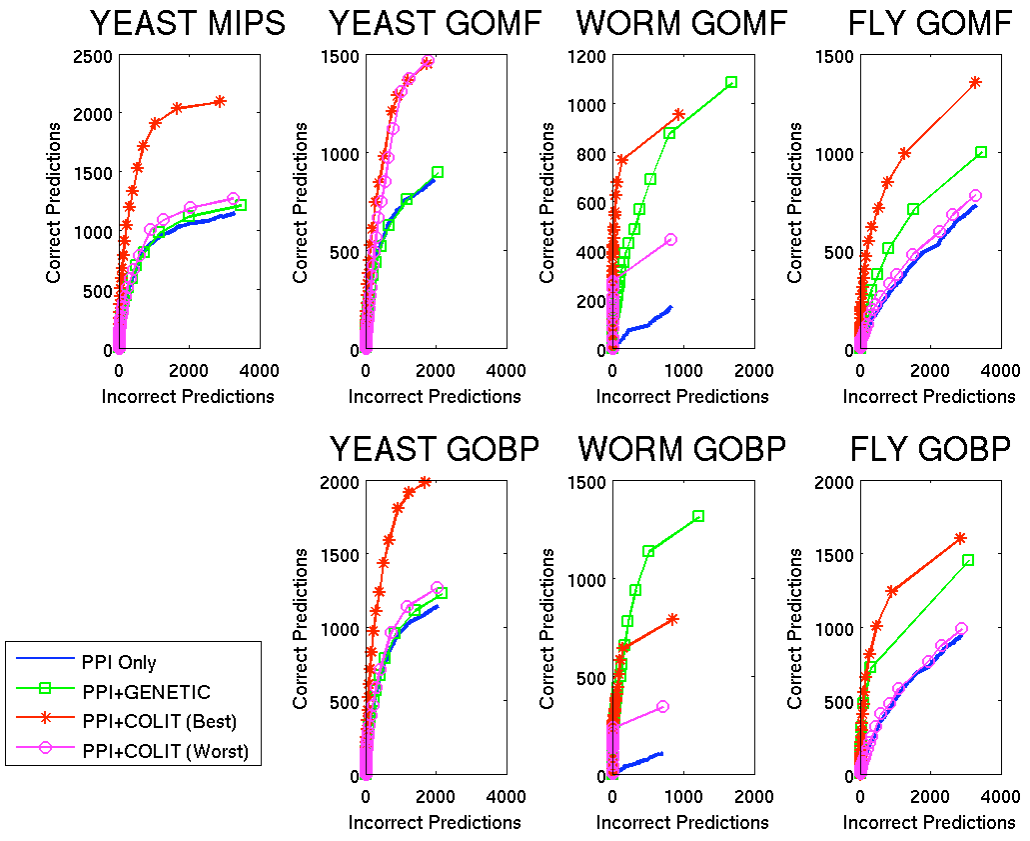
**Modified ROC curves for Functional Flow**. Number of proteins predicted incorrectly (FP) versus number of proteins predicted correctly (TP). Abbreviations: GOMF – GO SLIM Molecular Function; GOBP – GO SLIM Biological Process; PPI ONLY – only edges from experiments measuring protein-protein interactions, such as yeast two-hybrid and affinity precipitation; GENETIC ONLY – only edges from genetic assays, such as synthetic lethality studies; PPI+GENETIC – edges from both PPI and from genetic assays, such as synthetic lethality studies; PPI+COLIT – edges from both PPI and edges between proteins found by literature co-occurrence, where Best and Worst correspond to the best and worst combinations of threshold setting and co-occurrence measure, respectively (*c.f*. Figure 5).

**Figure 3:**
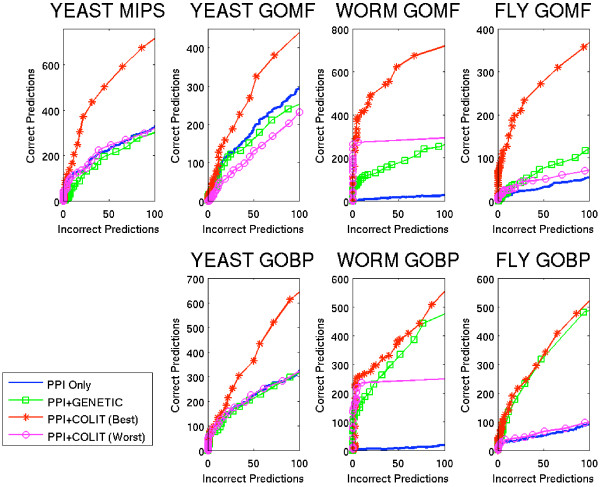
**Detailed Modified ROC curves for Functional Flow**. Number of proteins predicted incorrectly (FP) versus number of proteins predicted correctly (TP), for FP up to 100. Abbreviations as in Figure 2.

In all organisms, the performance of the Functional Flow algorithm was greatly boosted by the inclusion of co-occurrence data within the protein-protein interaction (PPI) network (Figure 2, Best and Worst correspond to combinations of co-occurrence measure and confidence threshold, chosen as explained below, yield the best and worst performance over all combinations). Most importantly, the difference was strong in the low false positive range, an area of particularly interest to biologists since false positives imply wasted experimental effort to validate the predictions (Figure 3). The strong data contribution to worm by non-PPI sources was reflected in large prediction performance differentials for Functional Flow, compared to the other two organisms (Figure 2 and 3).

Performance improvements of Functional Flow were not due merely to the presence of additional interactions, but rather to the type of relationship captured by the data sources. Supplementing the PPI network with a similarly-sized set of genetic interactions did not show as marked a performance gain as incorporating co-occurrence data (Figure 2 and 3). In nearly all cases, Functional Flow on the PPI+COLIT graphs performed better than when using PPI+GENETIC graphs, even though in most cases performance on the PPI+GENETIC graphs also showed modest to substantial improvement over using the PPI ONLY graphs. This suggests that the relationships implicit in literature abstracts prove more informative for function prediction than those captured by genetic screens.

**Figure 4:**
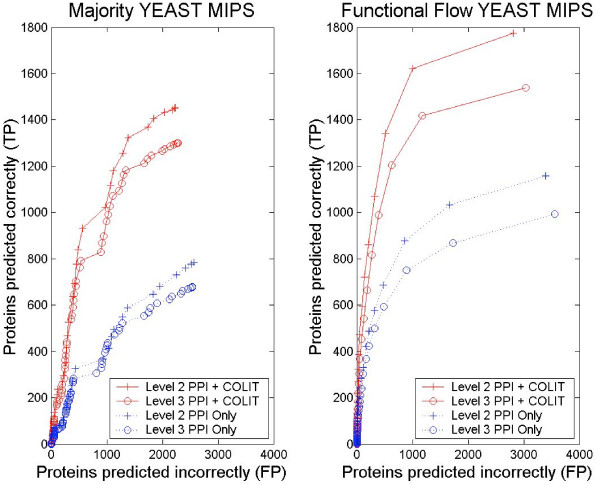
**Varying Annotation Granularity**. Performance as the level of annotation detail increases from Level 2 to Level 3 in the MIPS functional hierarchy. a) Majority, b) Functional Flow. Abbreviations: PPI ONLY – only edges from experiments measuring protein-protein interactions; PPI+COLIT – PPI edges combined with edges between proteins mentioned at least twice together in literature abstracts.

Co-occurrence data also boosted the performance for the local algorithm, Majority which uses the majority assignment of immediate neighbors method (Figure 4a). Including co-occurrence information did not change that Functional Flow outperformed the Majority method in all settings (data not shown). For the harder problem of predicting more finely grained function annotations, using co-occurrence data proved to be useful even when the number of annotation categories increased (Figure 4). When the granularity level of annotation was changed from Level 2 in the MIPS hierarchy to Level 3, both Majority and Functional Flow showed a drop in performance which reflected the increased difficulty of the prediction task. However, in all cases, prediction performance was better in the PPI+COLIT graphs versus the PPI ONLY graphs.

### Examining effect of threshold on co-occurrence

**Figure 5:**
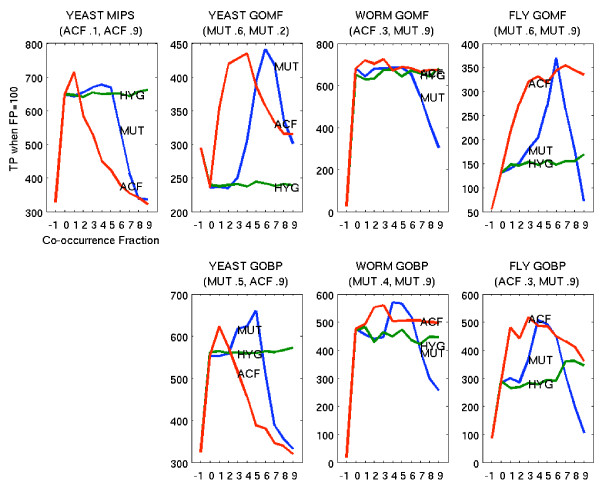
**Varying the Co-occurrence Threshold**. Relative performance of Functional Flow when varying the threshold used to define the co-occurrence interaction set. Shown is the number of true positives (TP) when the scoring threshold is set to yield 100 false positives (FP) (y axis). The values of the x-axis denote instances of Functional Flow on graphs combining PPI and the interaction sets for each corresponding setting of the co-occurrence threshold (x = -1 shows PPI ONLY and x = 0–9 denote PPI plus the datasets obtained using thresholds 0.0 to 0.9). The lines are annotated to denote the MUT, HYG and ACF metrics. The best and worst performers respectively, over all co-occurrence measure and all thresholds, are shown in parentheses below the plot title. These combinations appear as Best and Worst in Figures 2 and 3.

The performance boost offered by including literature data was seen regardless of the method for determining co-occurrence. The set of interactions offered in the co-occurrence data varied depending on a threshold. The best and worst combinations of co-occurrence measure and threshold (corresponding to Best and Worst in Figure 2 and 3) were chosen by examining the number of true positives when the number of false positives was fixed at 100, for PPI ONLY graphs and each setting of the threshold from 0 to 0.9 in increments of 0.1 (Figure 5). The addition of co-occurrence information for any measure, showed considerable improvement over the PPI ONLY graphs (marked as -1 on the x-axis), over most thresholds, with the exception of *HYG *and certain thresholds in yeast using Molecular Function. The worst often occurred for the 0.0 threshold, the simplest definition of co-occurrence, demonstrating the benefit of moving beyond a simple binary indicator of co-occurrence to methods based on confidence levels.

Among the three measures, the relatively flat performance of *HYG *reflected the fact that the measure essentially reduced to the binary indicator, causing the graphs to be nearly the same at any threshold. The sharp decrease in *MUT *reflected the behavior seen in the distributions (Figure 1) where a concentration of edge assignments mid-range resulted in a sharp decrease in graph size after 0.5. The general decrease at higher thresholds reflects that the combined PPI and *MUT *graphs contain fewer edges, which limits the ability of Functional Flow to exploit neighborhood information.

For *ACF*, the performance was generally high across all thresholds in worm and fly. Though the values in worm and fly might be inflated at higher thresholds due to large gene families in these organisms, *ACF *generally performed above *MUT*, despite a similar decrease in graph size. In yeast, the opposite was true where *MUT *performance at any fraction was generally higher than *ACF *regardless of the annotation source. This difference may be due to the lack of large families in yeast but may also include differences due to coverage and overlap of multiple PPI sources which occurred in yeast but not worm or fly. Overlap causes a strengthening of support for particular edges.

Overall, the *HYG *measure did not discriminate well between which co-occurrences were reliable or unreliable and therefore may not be ideal for this application. The *MUT *co-occurrence measure performs well for thresholds near 0.5, allowing a favorable compromise between edge reliability and graph connectiveness. However, the *ACF *measure shows comparable performance at lower thresholds, which allows a larger supplement of co-occurrence edges to the PPI graph.

## Discussion

Understanding the effect of supplemental data sources on a graph-theoretic function prediction algorithm involves asking three questions not typically asked – how readily available is this data source across species, how does changing species affect the source's impact, and what is the best way to include the source. Our results show that the amount of co-occurrence data extracted from the literature provides a significant fraction of the data available for an organism relative to the available protein-protein interaction data. Moreover, this additional resource offers interactions that are more likely to connect two functionally related proteins than the physical interaction data, which stems from the obvious bias of co-occurrence data toward well-studied proteins. The question then becomes how useful is co-occurrence data for unstudied proteins.

In our testing, function predictions is actually made for proteins with known functions, where the function is simply hidden to the algorithm. We do not alter the datasets in any additional way to reflect whether a protein's function is truly unknown. If a protein truly does not have a known function, a high-throughput protein-protein interaction is much more likely in our data than an abstract co-occurrence. In yeast, 96% of the uncharacterized proteins (using MIPS) have neighbors in the graph based solely on PPI data (3% have PPI and COLIT edges) versus 42% of the known proteins (45% have PPI and COLIT). Worm has a similar ratio with 80% PPI ONLY edges for the uncharacterized versus 53% PPI ONLY edges for the knowns, while fly follows with 93% PPI ONLY edges for the uncharacterized versus 60% PPI ONLY edges for the knowns.

The real concern then is how co-occurrence data can affect the prediction algorithm performance since the majority of true unknowns are solely connected to the rest of the graph through high-throughput protein-protein interactions. This question is particularly relevant in worm and fly with a larger percentage of truly uncharacterized proteins. The performance gain of the global Functional Flow algorithm over the local Majority algorithm precisely demonstrates how information can flow from a region of more well-characterized genes, where co-occurrence data is present, through regions surrounding the true unknowns and thus dominated by physical interaction data. In fact, the average minimum path distance between unknown proteins in yeast and proteins appearing in a least two Medline abstracts (thus likely to have a co-occurrence link) is 1.41 in yeast, 1.60 in worm and 1.387 in fly. Through the combination of protein-protein interactions and co-occurrence data, the neighborhood around unknown proteins is quickly connected to well-characterized nodes which global prediction algorithms can exploit.

The importance of the mix of protein-protein interaction data and co-occurrence data can also affect algorithm performance through individual edge weights. Since each source of evidence for an interaction is given a different weight based on its correspondence to shared function (Table 1), those interactions with multiple sources of evidence can have a variety of different weights in contrast to those edges added by a single source. Surprisingly, there was very little overlap between the protein-protein interaction data and the other sources (Table 3) which limits the ability of one source to reinforce evidence for the other. Regardless, the dramatic improvements in performance using co-occurrence data were seen as its edge weights were generally the highest for any data source, indicating strong concurrence with shared function. With respect to how changing the species affects performance, the relative performance of Function Flow on PPI networks augmented with *ACF *or *MUT *co-occurrence edges differed in yeast with respect to worm or fly (Figure 5). Also, the reliability estimates for the annotation sources suggested worm was different in that GO Molecular Function was a better characterization than GO Biological Process of the type of relationships represented by PPI and COLIT, while the opposite was true in yeast and fly (Table 1 and 2). The difference in worm may be attributed to the fact that over 65% of the GO annotations have the evidence code IEA (Inferred from Electronic Annotation) compared to 24% in fly and 0% in yeast. This disparity suggests that it is easier to infer Molecular Function from annotation sources than Biological Process. These organism-specific variations highlight the importance of benchmarking results in multiple organisms.

The comparison of co-occurrence measures is convoluted by the fact that for any given threshold, the graphs are not the same. Examples taken from the experiments show high *ACF *values capture cases missed by *MUT*, when a rarely studied protein is consistently associated with a highly-studied protein. These high *ACF *edges appear in the *MUT *≥ 0.5 threshold dataset and might explain the rising edge of the *MUT *curve in Figure 5 for thresholds lower than that. An alternative experiment would be to fix the graph topology, use the co-occurrence weight as the actual reliability of the edge (instead of using the reliability computed on the edge set at a given threshold) and evaluate function prediction performance in this setting. Such an experiment would further explore the benefits of using an asymmetric measure. Although co-occurrence data was still very useful when the annotation detail increased by one level on the MIPS hierarchy, it is possible that co-occurrences might become less useful if annotating proteins at the highest level of detail. Extremely specific relationships captured in the annotation source may not be represented at the same level of detail in the literature abstract text, and therefore not extracted as co-occurrence data. Some information such as the involvement of two given proteins in the same pathway might be as useful when looking at protein function described only by general categories. However, some information, such as shared structural features might be more useful when the function annotation source is in finer detail. It would be interesting to identify which sort of interactions a particular co-occurrence indicates and then examine which kind of abstract co-occurrences contribute the most at each level of detail in the annotation source. That sort of automatic categorization of interaction is currently at the limits of natural language processing systems; any such attempt still requires a great deal of hand curation. Regardless, whether by automatic or manual creation, the set of interactions suggested by co-occurrence which cannot be characterized as physical, genetic, structural or otherwise are the most interesting since these non-obvious links provide fruit for further study.

## Conclusion

Function prediction systems give uncategorized proteins likely annotations, helping biologists formulate testable hypotheses. We have demonstrated in at least two of these systems that performance can be greatly improved by including co-occurrence relationships drawn from abstracts. This increase in performance is not simply due to the presence of additional edges; supplementing protein-protein interactions with co-occurrence data outperforms supplementing with a comparably-sized dataset of genetic interactions. Following our success with graph-theoretic methods, machine learning approaches such as semidefinite programming based support vector machines may be able to use these co-occurrence graphs as well.

We have shown that co-occurrence data improves function prediction in a variety of circumstances. Our results hold across all three organisms of yeast, worm and fly despite significant differences in coverage and annotation for the sources. We demonstrated that the use of co-occurrence data can benefit from using a qualitative measure for determining interaction instead of a binary indicator and that generally a threshold of ACF ≥ 0.1 or *MUT *≥ 0.5 is a reasonable compromise regardless of organism or annotation source. Co-occurrence data provides a significantly large and readily available source of interaction data which, together with the guidelines and results reported here, will prove valuable especially for organisms in which protein-protein interaction data is sparse.

## Availability and requirements

WormBase: 

FlyBase: 

## Authors' contributions

APG initiated the research project and implemented the prediction algorithm. SML created the graphs and contributed to the analysis. WAB implemented the local copy of Medline used to find the co-occurrence information. LEH and DSG contributed to the formulation of the research plan and supervised the effort. All authors contributed to writing the manuscript.

## Supplementary Material

Additional file 1 Yeast co-occurrence graph where ACF is greater than .9. The complete PPI and co-occurrence network using ACF scoring at the highest threshold. Nodes correspond to yeast proteins and are colored by GO SLIM categories such that white nodes indicate Unknown Function. Edges between nodes *x *and *y *indicate *ACF*(*x*, *y*) > 0.9. Note that at this threshold, large clusters of like color indicate protein families. Click here for file

Additional file 2 Worm co-occurrence graph where ACF is greater than .9. The complete PPI and co-occurrence network using ACF scoring at the highest threshold. Nodes correspond to yeast proteins and are colored by GO SLIM categories such that white nodes indicate Unknown Function. Edges between nodes *x *and *y *indicate *ACF*(*x*, *y*) > 0.9. Note that at this threshold, large clusters of like color indicate protein families. Click here for file

Additional file 3 Fly co-occurrence graph where ACF is greater than .9. The complete PPI and co-occurrence network using ACF scoring at the highest threshold. Nodes correspond to yeast proteins and are colored by GO SLIM categories such that white nodes indicate Unknown Function. Edges between nodes *x *and *y *indicate *ACF*(*x*, *y*) > 0.9. Note that at this threshold, large clusters of like color indicate protein families. Click here for file
