# Supplementary material for: Improving protein function prediction methods with integrated literature data
Source: BMC Bioinformatics. 2008 Apr 15;9:198. doi: 10.1186/1471-2105-9-198 (PMC2375131; doi:10.1186/1471-2105-9-198)

Fly Interaction Network when ACF>0.9
colored by GO SLIM Molecular Function
(white nodes are unclassified)

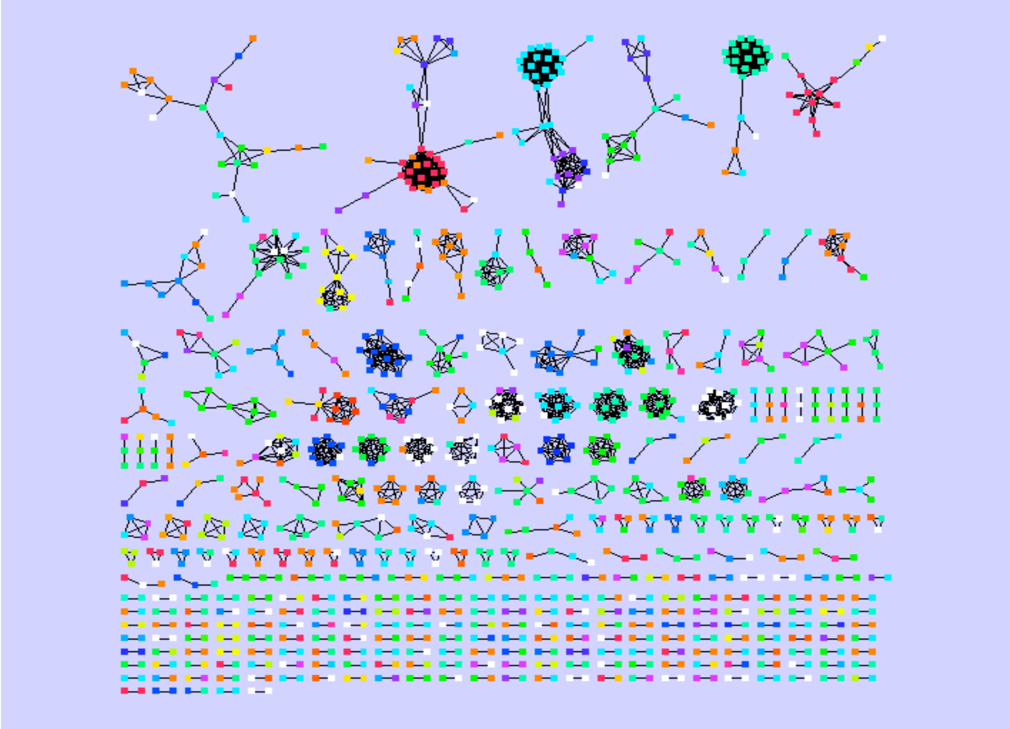

Fly Interaction Network when ACF>0.9
colored by GO SLIM Biological Process
(white nodes are unclassified)

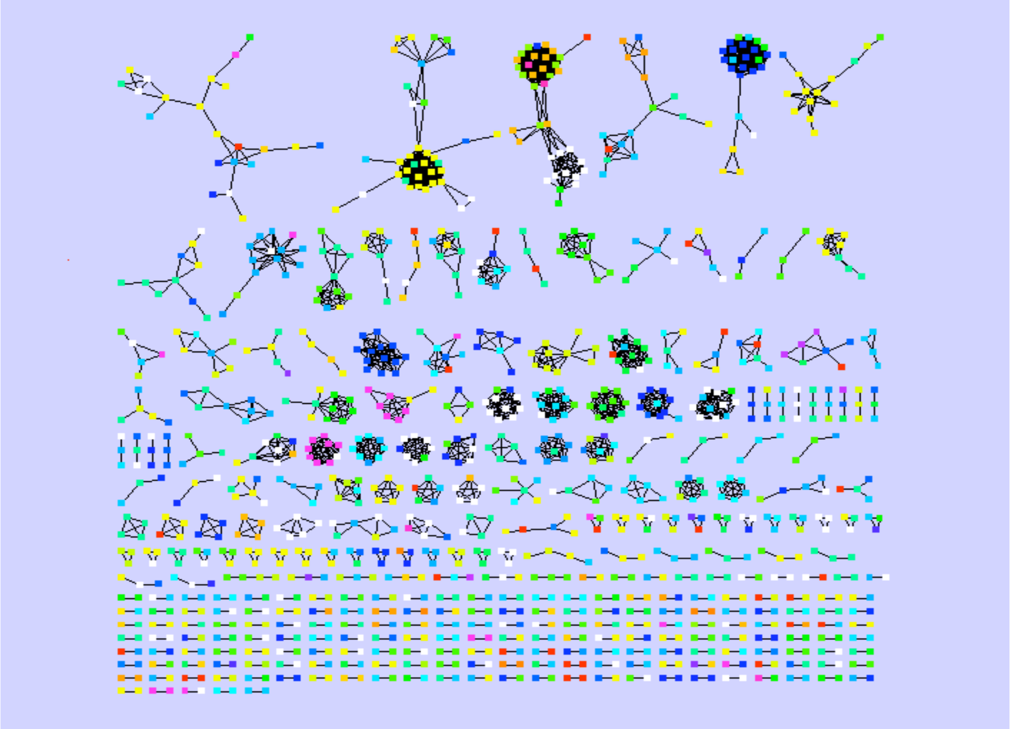

Supplement: Additional file 3 — Fly co-occurrence graph where ACF is greater than .9. The complete PPI and co-occurrence network using ACF scoring at the highest threshold. Nodes correspond to yeast proteins and are colored by GO SLIM categories such that white nodes indicate Unknown Function. Edges between nodes x and y indicate ACF(x, y) > 0.9. Note that at this threshold, large clusters of like color indicate protein families. [file 1471-2105-9-198-S3.pdf]
